# Disease of the Femur; Amputation at the Hip-joint, Recovery

**Published:** 1872-11

**Authors:** 


### Disease of the Femur; Amputation at the Hip-joint; Recovery.

The following case is of considerable interest, inasmuch as it is one of necrosis of the shaft of a large bone occurring without any suppuration whatever. The history pointed to a malignant affection, but a most careful examination of the bone after removal failed to detect such a condition, and showed without doubt a necrosis. For the notes of the case we are indebted to Mr. Parnell, house-surgeon.

J. B., aged twenty-two, a thin, emaciated young man, was admitted into St. Bartholomew's Hospital, under the care of Mr. Morrant Baker, on August 13, with disease of the left femur. The history is as follows: Ten weeks before admission he went to bed quite well, but on waking in the morning he felt great pain in his left thigh; on the next day he noticed that the thigh was swollen. This went on increasing for six weeks, when, as the patient was walking in his room, the bone broke spontaneously, and he fell down; and since then he has been unable to walk. On admission the left thigh was greatly swollen, the swelling being due to increased thickness of the bone. At about the middle third the bone was angularly curved, the prominence being outwards and forwards; and there was an inch and a half of shortening.

The case being diagnosed as malignant disease, amputation at the hip-joint was performed on August 15. Chloroform being administered, an abdominal tourniquet was applied over the lower part of the aorta. Antero-posterior flaps were then made by transfixion, the posterior flap being several inches longer than the anterior one. The femoral vessels were compressed by an assistant seizing the anterior flap as the knife cut outwards. There was very little blood lost, but many ligatures were necessary to secure the bleeding vessels. The surfaces of the flaps being washed with a carbolic acid solution, the wound was brought together by wire sutures, the ends of the ligatures hanging out at the extremity of the wound. Lastly, a large pad of lint soaked in carbolized olive oil was placed over the stump, which was then supported by means of a bandage.

On inspection of the removed limb, the muscles surrounding the bone were pale and appeared to be extensively infiltrated; the bone was thickened through the greater part of its extent, but markedly so at and below the seat of fracture, which was at the upper part of the middle third. On longitudinal section of the bone, the well-defined compact portion was seen to be surrounded by a firm casing about a quarter of an inch thick; here and there, however, the continuity of the compact tissue was interrupted. On a more careful examination made subsequently, it was found that the shaft of the bone was necrosed, and not the seat of malignant infiltration, as was at first supposed, and that the casing was made up of lymph thrown out by the periosteum and surrounding soft tissues.

Shortly after the operation, the patient was ordered fifteen drops of tincture of opium, with some brandy. In seven hours the temperature had gone up from 98.4° F. to 100°. On the day of the operation, and for the next two days, the patient was troubled with retching and vomiting.

Aug. 17th. The wound was dressed for the first time. Temperature, 101; pulse, 138.

20th. Wound suppurating freely. Temperature, 104.4°; pulse 120.

23d. Fifteen ligatures came away, and on the 24th five more were removed. Temperature, 100.2°.

From this date the temperature gradually descended to the normal, the appetite being good throughout.

Sept. 5th. The wound is rapidly healing; the last ligature came away to-day.

The local treatment has been throughout the thorough sluicing of the interior of the stump with weak permanganate of potash lotion twice daily, by using a rubber tube, with an elastic catheter to be introduced within the wound attached to one end, as a syphon from a basin containing the lotion.—*Lancet*, Sept. 21, 1872.—(*Med. News and Library.*)

